# Psychometric validation of the Pyruvate Kinase Deficiency Diary and Pyruvate Kinase Deficiency Impact Assessment in adults in the phase 3 ACTIVATE trial

**DOI:** 10.1186/s41687-023-00650-3

**Published:** 2023-11-09

**Authors:** David A. Andrae, Rachael F. Grace, Adrian Jewett, Brandon Foster, Robert J. Klaassen, Sam Salek, Junlong Li, Feng Tai, Audra N. Boscoe, Erin Zagadailov

**Affiliations:** 1 Endpoint Outcomes, Boston, MA USA; 2 Dana-Farber/Boston Children’s Cancer and Blood Disorders Center, Harvard Medical School, Boston, MA USA; 3 Division of Hematology/Oncology, Children’s Hospital of Eastern Ontario, Ottawa, ON Canada; 4 School of Life and Medical Sciences, University of Hertfordshire, Hatfield, UK; 5 Agios Pharmaceuticals, Inc., 88 Sidney Street, Cambridge, MA 02139–4169 USA

**Keywords:** Health-related quality of life, Psychometric validation, Pyruvate kinase deficiency, Pyruvate Kinase Deficiency Diary (PKDD), Pyruvate Kinase Deficiency Impact Assessment (PKDIA)

## Abstract

**Background:**

Pyruvate kinase (PK) deficiency is a rare hereditary disorder characterized by chronic hemolytic anemia and serious sequalae which negatively affect patient quality of life. This study aimed to psychometrically validate the first disease-specific patient-reported outcome (PRO) instruments: the 7-item PK Deficiency Diary (PKDD) and 12-item PK Deficiency Impact Assessment (PKDIA), designed to assess signs, symptoms, and impacts of PK deficiency in patients enrolled in the ACTIVATE global phase 3 study of mitapivat versus placebo (NCT03548220).

**Methods:**

All validation analyses for the PKDD and PKDIA were performed on blinded data, with analyses on item integrity, scoring, reliability, and validity conducted on data from screening and baseline. Completion rates and baseline response distributions were characterized using descriptive statistics. Item response modelling was used to inform a weighted scoring system. Reliability was assessed by internal consistency and test–retest reliability; and validity by convergent and known-groups analyses.

**Results:**

Of the 80 adults enrolled, baseline data were available for 77 (96.3%) and 78 (97.5%) patients for the PKDD and PKDIA, respectively. Item responses skewed right, indicating that mean values exceeded median values, especially for items utilizing a 0–10 numeric scale, which were subsequently recoded to a 0–4 scale; 4 items were removed from the PKDIA due to redundancy or low relevance to the trial population. Both the PKDD and PKDIA demonstrated high internal consistency (McDonald’s coefficient ω = 0.86 and 0.90, respectively), test–retest reliability (intra-class coefficients of 0.94 and 0.87, respectively), and convergent validity with other PROs (linear correlation coefficients [|r|] between 0.30–0.73 and 0.50–0.82, respectively).

**Conclusions:**

The findings provide evidence of validity and reliability for the PKDD and PKDIA, the first disease-specific PRO measures for PK deficiency, and can therefore increase understanding of, and more accurately capture, the wider impact of PK deficiency on health-related quality of life.

*Trial registration* ClinicalTrials.gov, NCT03548220. Registered June 07, 2018; https://www.clinicaltrials.gov/ct2/show/NCT03548220.

**Supplementary Information:**

The online version contains supplementary material available at 10.1186/s41687-023-00650-3.

## Plain language summary

Pyruvate kinase (PK) deficiency is a rare genetic blood disorder with a wide range of signs and symptoms that may have a negative impact on patients’ quality of life. Patient-reported outcome (PRO) instruments are tools that assess how a disease affects a patient from the patient’s perspective. These instruments must go through a validation process to make sure they truly capture the patient’s experience with their condition or its treatment. This study aimed to validate two new PRO instruments in adult patients enrolled in the ACTIVATE clinical trial (NCT03548220), where patients with PK deficiency received the drug mitapivat or a placebo. These two new PRO instruments are the first to be developed specifically for PK deficiency: the PK Deficiency Diary (PKDD), a daily diary that asks 7 questions to measure the core signs and symptoms of PK deficiency, and the PK Deficiency Impact Assessment (PKDIA), a weekly questionnaire with 12 questions to assess the impact of PK deficiency on a patient’s life. The results of this study showed that the PKDD and PKDIA properly and reliably measured the signs, symptoms, and impacts of PK deficiency that they aimed to capture. These findings indicate that the PKDD and PKDIA are the first validated PROs specifically for PK deficiency and can help improve the understanding of the impact of PK deficiency on patients’ quality of life.

**Supplementary Information:**

The online version contains supplementary material available at 10.1186/s41687-023-00650-3.

## Introduction

Pyruvate kinase (PK) deficiency is a rare, hereditary, non-spherocytic hemolytic anemia [1, 2], with an estimated diagnosed prevalence in Western populations of 3.2–8.5 cases per million [3]. PK deficiency is caused by mutations in the *PKLR* gene which encodes pyruvate kinase R (PKR) in red blood cells (RBCs), an enzyme that is responsible for the final step of glycolysis and is critical for the maintenance of RBC energy levels and cell integrity [4–7]. Defective PKR results in chronic hemolysis with premature breakdown of RBCs leading to acute symptoms, as well as long-term complications and consequences that include, but are not limited to, iron overload, fatigue, osteoporosis, and jaundice [1, 2, 8, 9]. Consequently, PK deficiency and its sequalae result in a profound negative impact on patient health-related quality of life (HRQoL) [10].

A qualitative study of HRQoL in patients with PK deficiency documented a wide range of signs, symptoms, and disease impacts, with those related to physical function, such as a lack of energy (tiredness, fatigue, shortness of breath), and to appearance, such as jaundice, reported by patients as particularly burdensome [9]. These results highlight the need for relevant, reliable, and valid disease-specific methods for the assessment of patient-reported outcomes (PROs) for PK deficiency [11]. Subsequently, two specific PRO instruments were established to measure the signs, symptoms, and impact of the disease on adult patient HRQoL. The PK Deficiency Diary (PKDD) is a 7-item daily evening diary survey to measure the core signs and symptoms of PK deficiency, and the PK Deficiency Impact Assessment (PKDIA) is a weekly 12-item questionnaire to assess the impact of PK deficiency (Table 1) [12]. The development of these novel instruments was informed by insights from 21 concept elicitation interviews and 20 cognitive debriefing interviews with patients in the United States, Germany, and the Netherlands [9, 12], in accordance with the United States Food and Drug Administration’s (US FDA) PRO guidance [13, 14]. The details of instrument development have been described previously [12].

**Table 1:** Summary of PKDD and PKDIA items^a^

	Description	Score range
PKDD item^b^		
1	Describe how tired you were at its worst today	0–10
2	Describe how tired you felt after finishing your daily activities (e.g., work, social, leisure, physical or household activities) today	0–10
3	Describe your jaundice (how yellow your eyes and/or skin appeared) when you looked in the mirror today	0–4 (None–very severe)
4	Describe your bone pain at its worst today Option: I have never experienced bone pain	0–10
5	Describe your shortness of breath during moderate (e.g., walking on an incline or upstairs) physical activity you did today Option: I avoided this activity because it was too difficult for me to do moderate physical activity Option: Not applicable, because I did not have the opportunity to do moderate physical activity	0–10
6	Describe your energy level at the beginning of your day (after being awake for one hour)	0–10
7	Describe your energy level at the end of your day (right now)	0–10
PKDIA item^c^		
1	Start things you wanted to get done	0–10
2	Finish things you wanted to get done	0–10
3	How often were you bothered by your appearance because of your PK deficiency over the past 7 days?	0–10
4	How often did you get unwanted attention because of your PK deficiency over the past 7 days?	0–10
5	How often did your PK deficiency interfere with your ability to do household activities (e.g., chores, cleaning, laundry) over the past 7 days?	0–10
6	How often did a lack of energy due to your PK deficiency interfere with participating in social activities (e.g., doing something together with friends) over the past 7 days?	0–10
7	How often did your PK deficiency interfere with leisure activities (i.e., hobbies or things you do for fun in your free time) over the past 7 days?	0–10
8	How often did you feel your relationships with friends or family were negatively affected because of your PK deficiency over the past 7 days?	0–10
9a	Did you work or go to school over the past 7 days? Option: Yes Option: No, because it was too difficult for me to go to work or school Option: No, because I am not currently working or in school for reasons unrelated to my PK deficiency	Yes/no
9b	*[If yes to Question 9a]* How often did your PK deficiency interfere with your ability to perform to your full potential at work or school over the past 7 days?	0–10
10	How often did you have difficulty concentrating because of your PK deficiency over the past 7 days?	0–10
11a	Did you perform moderate (e.g., walking on an incline or up stairs) physical activity over the past 7 days? a. Yes b. No, because it was too difficult for me to do moderate physical activity c. No, because I did not have the opportunity to do moderate physical activity	a/b/c
11b	*[If yes to Question 11a]* How often did you have difficulty performing moderate (e.g., walking on an incline or up stairs) physical activity because of your PK deficiency over the past 7 days?	0–10
12	How much additional rest or sleep did you feel you needed because of your PK deficiency over the past 7 days?	0–4 (None–a lot)

^a^Ownership of the PKDD and PKDIA resides with Agios

^b^Higher scores are worse for all items except 6 and 7

^c^Higher scores are worse for all items except 4

PKDD items 1 and 2 were graded from “0—not at all tired” to “10—extremely tired”, items 4 and 5 from “0—none” to “10—worst possible”, and items 6 and 7 from “0—low energy” to “10—high energy”. PKDIA items 1 and 2 were graded from “0—not at all difficult” to “10—extremely difficult”, items 3–8, 9b, 10, and 11b were graded from “0—none of the time” to “10—all of the time”

*PK* pyruvate kinase, *PKDD* Pyruvate Kinase Deficiency Diary, *PKDIA* Pyruvate Kinase Deficiency Impact Assessment

The use of appropriate qualitative interviews is an important contributor to the development of patient-centric PROs, ensuring concepts that are meaningful to patients are captured. Assessment of the measurement properties of PRO instruments is also vital to ensure they are reliable, valid, and responsive tools for the assessment of the signs, symptoms, and impact of PK deficiency. Here we report the in-trial psychometric validation of the PKDD and PKDIA. The objectives of these analyses were to establish the quantitative structure, reliability, and validity of the instruments using blinded data from patients enrolled in the ACTIVATE clinical trial (NCT03548220).

## Methods

### Study design and participants

Details of the ACTIVATE trial have been previously published [15]. ACTIVATE was a randomized, multicenter, double-blind, placebo-controlled, phase 3 study in which 80 adults with PK deficiency who were not regularly transfused (≤ 4 transfusion episodes in the previous year and no transfusion episodes within 3 months before the first day of study treatment) were randomized 1:1 to receive oral mitapivat or placebo twice a day, stratified by average screening hemoglobin levels (< 8.5 vs. ≥ 8.5 g/dL) and *PKLR* gene mutation category (missense/missense vs. missense/non-missense). Patients were screened for up to 42 days before randomization, after which eligible patients received 5 mg mitapivat or placebo for 4 weeks as a starting dose, followed by 2 potential sequential dose level increases to 20 mg and 50 mg BID at Weeks 4 and 8, respectively, dependent on safety and efficacy assessments. After this 12-week dose-optimization period, dose regimens were maintained through a 12-week fixed-dose period [15]. Study baseline was defined as the seven days leading up to randomization for the PKDD, and as the last non-missing administration of the instrument for each patient prior to randomization for the PKDIA.

### Patient-reported outcome measures

The PKDD is a self-administered, daily, 7-item PRO measure of the core signs and symptoms of PK deficiency in adults, with a 24-h recall period constituting the same day as administration (Table 1). Of the seven items, six have response options that are coded to an 11-point numerical rating scale (NRS) that focuses on severity of symptoms (e.g., tiredness and bone pain), and the remaining item is rated on a 5-step verbal descriptor scale (VDS), which asks patients to describe the severity of their jaundice. The PKDD assessed three groupings of signs and symptoms, those which related to energy (items 1, 2, 6, and 7), to appearance (item 3), and to anemia (items 4 and 5; Table 1). In ACTIVATE, the PKDD was collected daily from the first day of the 42-day screening period through the end of the fixed dose period at Week 24 (Additional file 1: Table S1).

The PKDIA is a 12-item PRO measure, which assesses the impacts of PK deficiency experienced by adults on their HRQoL (e.g., feeling bothered by appearance, impact on the ability to do household activities and on moderate physical activity etc.). The recall period for all PKDIA questions is the past seven days. Items 1–8, 9b, 10, and 11b have response options coded to an 11-point NRS. Items 9 and 11 are two-part items where part *a* coded responses as to whether the topics were applicable to the patients and part *b* is only recorded if patients answered *Yes* to the part *a* items. Item 12 is rated on a 5-step VDS. The PKDIA assessed seven groupings of the impact of PK deficiency, relating to daily living (items 1, 2, and 5), appearance (item 3), socializing (items 4, 7, and 8), leisure activities (item 6), work or school (item 9), cognition (item 10), and physical (items 11 and 12; Table 1). The PKDIA was collected on the first day of screening, at weekly intervals (up to 6 weeks) during screening, and then every 4 weeks thereafter through the dose optimization and fixed dose periods (Additional file 1: Table S1).

To support convergent and discriminant validity analyses of the PKDD and PKDIA, additional PROs were employed that were hypothesized to be part of the nomological network of constructs identified as important to patients using Cronbach’s method, built on the concepts of Campbell and Fiske [16, 17]. The following PRO measures implemented in the ACTIVATE study (Additional file 1: Tables S1 and S2) were utilized:

- The European Quality of Life Five-Dimensional Descriptive System (EQ-5D-5L), that measures mobility, self-care, usual activities, pain/discomfort, and anxiety/depression;
- The Functional Assessment of Cancer Therapy-Anemia (FACT-An), that assesses fatigue and anemia-related concerns;
- The mental health and physical functioning component summaries (MCS and PCS, respectively) of the 12-item short form health survey version 2.0 (SF-12v2);
- The patient global impression of severity (PGIS), a disease-specific, single-item measure that assesses the patient-reported severity of the effect of PK deficiency over one day, rated on a 5-point Likert scale VDS (severity categories: 0 = not a lot; 1 = a little; 2 = moderately; 3 = a lot; and 4 = very much).

The PGIS rated at baseline was also used in the assessment of known-groups validity for the PKDD and PKDIA. For collection of all PRO data, patients were provided with an electronic diary (Signant Health ePRO platform) at screening to record responses to PRO measures within the appropriate recall timeframe throughout the trial.

### Psychometric analyses

Psychometric analyses were conducted to evaluate and establish the quantitative structure, validity, and reliability of the PKDD and PKDIA scores. Blinded data collected at screening and baseline of the ACTIVATE trial were used. The analyses were performed using the “psych” (version 2.0.12 [18] or higher) and the “mirt” packages (version 1.33.2) [19], within R (version 3.6.3 or higher) [20].

### Quantitative structure

As a precursor to estimating statistical models, item response distributions were assessed for both item sparseness and floor or ceiling effects, analyses which function as leading indicators of potentially problematic issues that can be further evaluated using more specific investigations of item functioning in the downstream item-response theory (IRT) modeling [21, 22]. Item calibration was performed at baseline administrations (which constituted the seven days leading up to randomization for the PKDD, and the last non-missing administration of the instrument for each patient prior to randomization for the PKDIA). The proportion of missing data was also assessed (reported as percentage of missing diaries out of total contributed), and the quality of completion for the PKDD and PKDIA was defined as the average sample present-data over the duration of collection. Item response distributions for each item of the PKDD and PKDIA were characterized descriptively. Floor and ceiling effects were examined, both defined for items with *k* response categories (e.g., for an 11-point NRS, *k* = 11), if the most extreme category response percentages exceeded $\frac{100}{{k}}\%$. This assessment was done to identify items that may have posed issues to the estimated psychometric models that followed; items with high rates of missingness or floor or ceiling effects were flagged for further investigation.

The rate of completion for the PKDD and PKDIA measures at each assessment was reported for pre- and post-treatment periods to characterize the observed missing data and completion patterns. The overall response patterns were evaluated initially to determine any unusual answering that may indicate a problematic response pattern (such as levels of non-completion, consistent misinterpretation of the item question, the frequency of maximum/minimum item scoring, or large within-assessment variability). The frequency and percentage of each response option for each item on the PKDD and PKDIA measures were assessed in the sample across administrations (i.e., approximately daily [PKDD] or weekly [PKDIA] for the duration of participant enrolment) were evaluated. Spearman correlations were used to detect excessively strong or weak inter-item relationships to inform the presence of unstable inter-item relationships that could affect the model-fitting process. Items consistently showing |r|< 0.15 or |r|> 0.85 were used to determine problematic items and were flagged for further investigation [23].

IRT modelling, specifically the heterogeneous graded response model (GRM) [24], was used to determine how items aggregated together to define relevant constructs measured by the PKDD and PKDIA, the quality of response categories, and to optimize scoring algorithms. Model fit indices and statistics designed to evaluate given structures were used to determine the optimal number of domains needed to explain the PKDD and PKDIA data. To assess the dimensionality of the scale, several full-information maximum likelihood models were fitted. Full-information maximum likelihood was used to ensure the most accurate estimation of the models, as this approach considers all data collected, including missing data. Focus was placed on unidimensional models for each of the PKDD and PKDIA, as the likelihood of stable sub-scales was low given the relatively small number of items in each instrument [25]. To determine the most appropriate number of factors to extract, model fits from competing models were evaluated with the C_2_ statistic for absolute fit [26], the root mean squared error of approximation (RMSEA), comparative fit index (CFI), Tucker–Lewis index (TLI), and standardized root mean square residual (SRMR). Due to sparse response patterns observed for the PKDD, to aid in determining if model parameters exhibited factor invariance longitudinal IRT models were fit over Days − 7 to − 1. The model parameters from each day were then used as plausible values to estimate marginalized parameters for the baseline week. This set of estimates was then used to score the PKDD for each administration. Modelling of the PKDIA employed the last-reported administration per respondent prior to randomization. The 11-level item NRS responses for both the PKDD and PKDIA were evaluated for appropriate response scaling, to assess whether collapsing of response options was indicated.

IRT model fits for the PKDD and PKDIA were used to inform a weighted scoring algorithm which translated model-based sum scores to a T-score metric using expected a priori (EAP) scoring methods within the “mirt” version 1.33.2 packages’ "fscores" function [19, 27]. This was done to improve precision from IRT-based scoring whilst allowing a transformation without the need for specialist software to compute EAP scores. As the PKDD was recorded each day, daily PKDD scores were averaged over a seven-day period if at least 4 days of data were available in a week (otherwise the score was treated as missing). The T-score method was selected as it has known characteristics (normally distributed data with a mean of 50 and SD of 10), has been utilized in similar assessment measures [28], and it generates positive scale values, which alleviates potential interpretation issues with Z-score values.

#### Reliability

Internal consistency for the PKDD and PKDIA was measured via empirical internal reliability estimates of the model using McDonald’s coefficient ω [29]. Test–retest reliability was evaluated using the *ICC* (2,1) (two-way mixed effects intraclass correlation coefficient) across a 1-week retest period, using scores from screening and baseline [30].

#### Validity

Convergent validity (convergent construct validity testing a pre-defined hypothesis) was assessed by determining the extent to which scores on a PRO under development correlate with PGIS and FACT-An, which measure the same or related constructs, with strong relationships expected. In contrast, discriminant validity was assessed by determining relationships between PKDD and PKDIA scores and EQ-5D-5L, and MCS-12 and PCS-12 from the SF-12v2, which are thought to be unrelated to the constructs measured by the PKDD and PKDIA, with weak relationships expected. Convergent and discriminant validity were assessed using Spearman correlations. Cohen’s interpretation of r as an effect size was also employed, with correlation coefficients interpreted as small (0.1 ≤|r|), medium (0.3 ≤|r|) or large (0.5 ≤|r|) [31].

Known-groups validity was assessed to evaluate the ability of the PKDD and PKDIA to discriminate between distinct groups when a difference between them is expected and was assessed using a linear model and PKDD and PKDIA data at baseline, with known-groups defined by baseline PGIS score.

## Results

A total of 80 patients were enrolled in the ACTIVATE phase 3 study. Baseline demographics and disease characteristics have been previously reported [15]. In brief, mean ± SD hemoglobin levels were 8.6 ± 1.0 and 8.5 ± 0.8 g/dL in the mitapivat and placebo groups, respectively, ≥ 70% of patients had splenectomy, cholecystectomy, or both, > 50% of patients had decreased bone mineral density, and patients in both groups had elevated ferritin levels (747.9 ± 1116.2 and 688.0 ± 605.2 μg/L in the mitapivat and placebo groups, respectively). PKDD data were available for 77 (96.3%), 77 (96.3%), and 73 patients (91.3%) at baseline, Week 12, and Week 24, respectively, while PKDIA data were available for 78 (97.5%), 70 (87.5%), and 67 patients (83.8%), respectively.

### Quantitative structure—PKDD

**Fig. 1:**
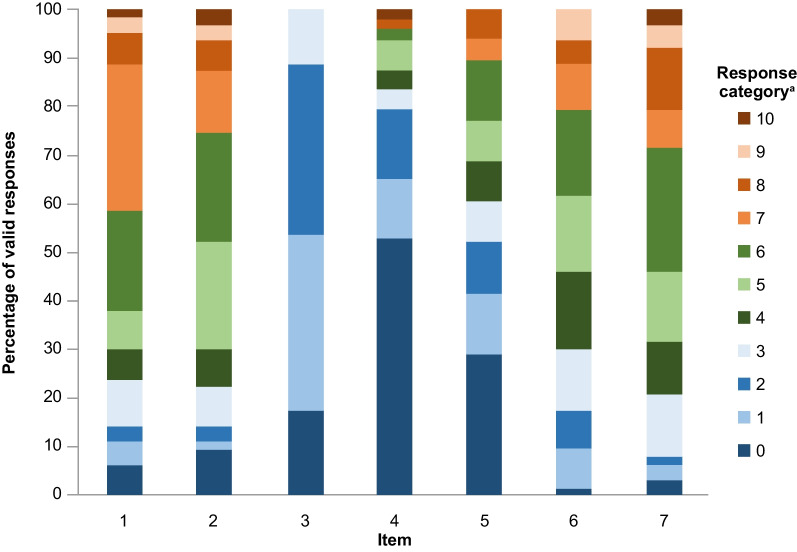
PKDD item response distributions at baseline. ^a^Items 1, 2, and 4–7 have response options coded to an 11-point numerical rating scale. Item 3 is rated on a 5-step verbal descriptor scale so only responses from 0 to 4 are available. See Table 1 for a full summary of PKDD items. In brief, item 1, tiredness at its worst; 2, tiredness after daily activities; 3, jaundice; 4, bone pain; 5, shortness of breath; 6, energy at the beginning of the day; 7, energy at the end of the day. *PKDD* Pyruvate Kinase Deficiency Diary

**Fig. 2:**
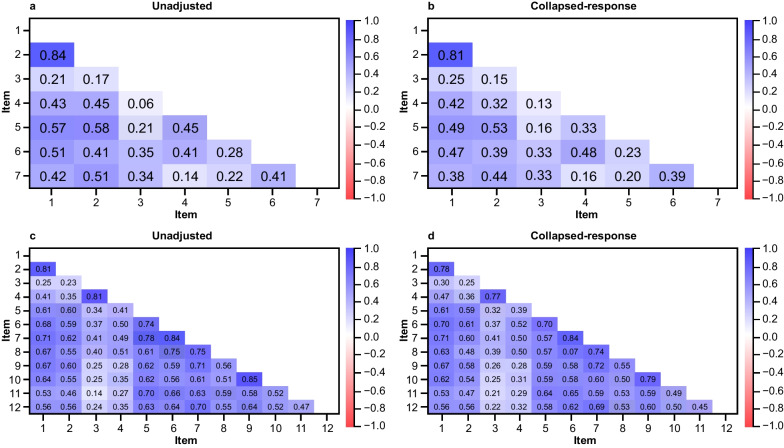
Inter-item Spearman correlation matrices for PKDD (**a**, **b**) and PKDIA (**c**, **d**) items at baseline. For the PKDD, the scoring values for Items 6 and 7 were inverted for consistency, to ensure that higher scores represented greater disease burden for all items. *PKDD* Pyruvate Kinase Deficiency Diary, *PKDIA* Pyruvate Kinase Deficiency Impact Assessment

Response distributions for the PKDD data were skewed towards the right, i.e., respondents tended to report scores at the lower ends of the response options (low severity). This was observed particularly for Items 3 and 4 (Fig. 1; Additional file 1: Table S3) which may explain a lower-than-expected correlation between these two items. Items 4 and 5 contained optional response categories where the item topic may not be relevant or applicable to the patient; where this was the case responses were recorded as “missing data”. Response distributions were similar when assessed with or without missing data (Fig. 1; Additional file 1: Table S3). Inter-item correlation values fell within the target range except for Items 3 and 4 (Fig. 2a), which had the lowest correlation (see Table 1 for summary of item descriptions). For IRT modeling, the six 11-point items (items 1, 2, and 4–7) were collapsed so that all items had five coded values, with 0 representing low scores and 4 high scores. These adjustments showed similar correlations to the unadjusted PKDD items (Fig. 2b), and all 7 items were retained in the scoring algorithm.

The GRM demonstrated excellent model fit (C_2_ = 11.81, degrees of freedom [df] = 14, *p* = 0.622; RMSEA < 0.01 [95% CI < 0.001, 0.132], SRMR = 0.15, TLI = 1.00, CFI = 1.00), with only the SRMR falling slightly above the recommended level (0.10), indicating some difference between observed and predicted responses (Additional file 1: Table S4). Additionally, for the estimated latent distribution of scores (*θ*) no mean values were greater than 2 standard error values from 0 for means, and 1 standard error value for variances, which demonstrated *θ* was relatively stable during the baseline week, as estimated by the GRM (Additional file 1: Table S5).

Based on this model, a weighted scoring algorithm was developed for the PKDD and implemented using the EAP sum score conversion tables. The scoring algorithm was as follows. Within each day, scores within respective domains were summed using the collapsed response categories. These sum scores were then converted to T-score transformed EAP sum scores using a “look-up” table, and then these daily scores were averaged over a seven-day period if at least 4 days of data were available in a week (otherwise the score was treated as missing) (Additional file 1: Table S6). This approach generated a PKDD T-score range from 25 to 76, where higher T-scores represented greater disease burden.

### Quantitative structure—PKDIA

**Fig. 3:**
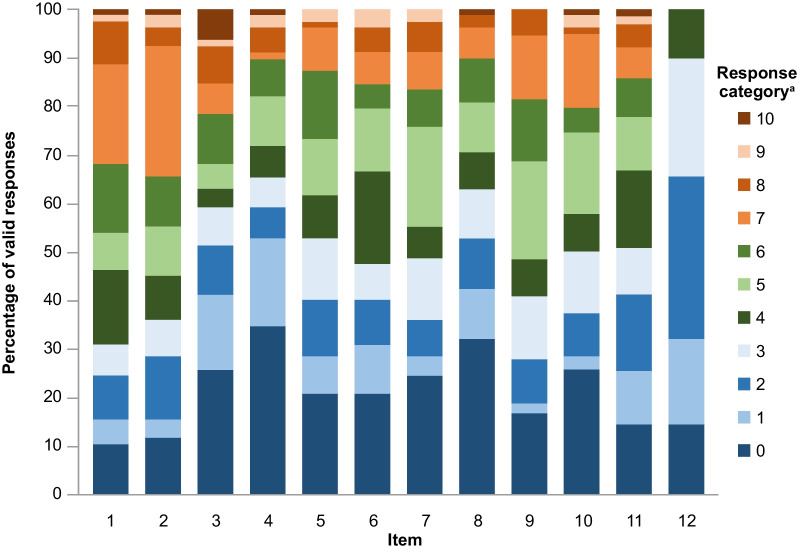
PKDIA item response distributions at baseline. ^a^Items 1–8, 9b, 10, and 11b have response options coded to an 11-point numerical rating scale. Items 9 and 11 are two-part items where part *a* coded responses as to whether the topics were applicable to the patients and part *b* is only recorded if patients answered *Yes* to the part *a* items. Item 12 is rated on a 5-step verbal descriptor scale so only responses from 0 to 4 are available. See Table 1 for a full summary of PKDIA items. In brief, item 1, starting things you want to get done; 2, finishing things you want to get done; 3, bothered by appearance; 4, unwanted attention; 5, impact on household activities; 6, impact on social activities; 7, impact on leisure activities; 8, impact on social relationships; 9, impact on work/school; 10, concentration; 11, physical activity; 12, additional sleep or rest. *PKDIA* Pyruvate Kinase Deficiency Impact Assessment

Response distributions were similarly skewed to lower ratings for the PKDIA (Fig. 3; Additional file 1: Table S7), so the same collapsed-response approach used for the PKDD was employed to adjust 11-point items (items 1–11) to five coded values. Inter-item correlations all fell within the target range except for the correlation between Items 3 and 11 (r = 0.54) (Fig. 2c), and again a similar pattern of correlations to the unadjusted items was observed (Fig. 2d).

The initial GRM for baseline item responses was a poor model fit (C_2_ = 114.26, df = 54, *p* < 0.001, RMSEA = 0.159 [95% CI 0.117, 0.200], SRMR = 0.18, TLI = 0.89, CFI = 0.91). Following evaluation, four items (1, 3, 4, and 9; see Table 1 for summary of items) were identified that were either less relevant to the ACTIVATE trial population or did not contribute unique information due to skewness or redundancy. These were related to daily living (item 1), appearance (item 3), social activity (item 4), and attendance at work/school (item 9). Item 1 had high redundancy with and less preferable response parameters than item 2; items 3 and 4 had very large floor effects (41% and 53% of responses were 0 in the reduced item set, respectively) and had high inter-item correlation (r = 0.77), which indicated misfit within the model and possible irrelevance to the patient group; item 9 showed a high rate of irrelevance to patients, with 27% reporting no issues with performance at work or school. The final model removed these four items from the scoring system, although they were kept within the questionnaire for informational purposes. The remaining 8-item set resulted in a GRM model with excellent fit (C_2_ = 22.28, df = 20, *p* = 0.326; RMSEA = 0.043 [95% CI < 0.001, 0.120]) (Additional file 1: Table S4). A scoring algorithm was then developed converting item sums to T-scores (Additional file 1: Table S8), which resulted in an observed PKDIA T-score range from 30 to 76, where higher T-scores represented greater disease burden.

### Reliability and validity—PKDD

**Fig. 4:**
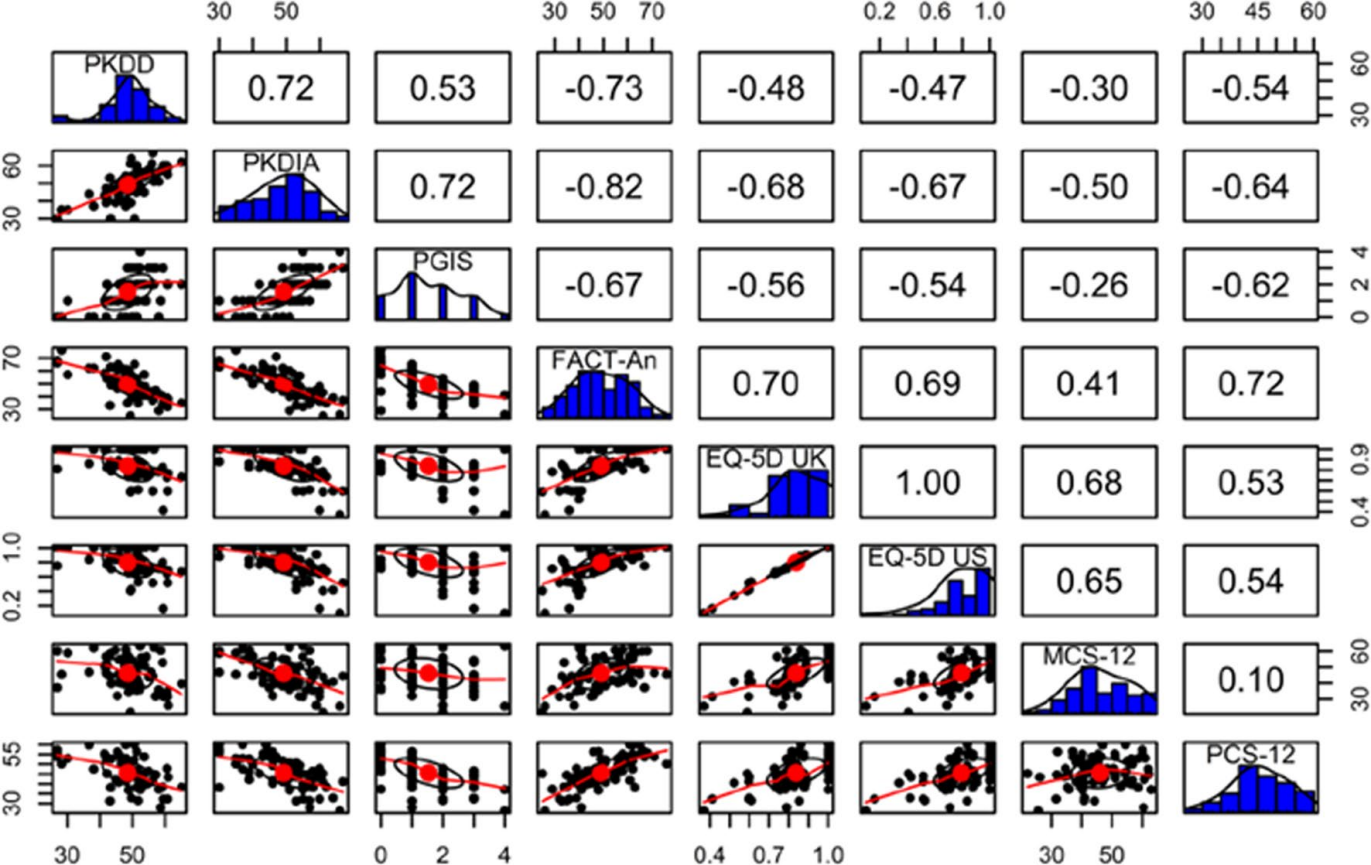
Spearman correlations between PKDD, PKDIA, and other co-validating scores at baseline. *EQ-5D-5L UK* United Kingdom-normed European quality of life 5-dimension score, *EQ-5D-5L US* United States-normed European quality of life 5-dimension score, *FACT-An* Functional Assessment of Cancer Therapy Anemia, *MCS-12* 12-item mental component summary, *PCS-12* 12-item physical component summary, *PGIS* patient global impression of severity, *PKDD* Pyruvate Kinase Deficiency Diary, *PKDIA* Pyruvate Kinase Deficiency Impact Assessment

Internal consistency was high for the PKDD, ω = 0.86, above the usual criterion of ω = 0.70. Test–retest reliability was excellent [30], with an *ICC* (2,1) value of 0.94. Assessment of validity demonstrated medium correlations between baseline PKDD and PKDIA scores (|r|= 0.72); correlations between PKDD and other scores were small to medium (|r|= 0.30–0.73) (Fig. 4). The strongest correlation was observed between the PKDD and the FACT-An Additional Concerns Subscale (|r|= 0.73), showing evidence for convergent validity (Fig. 4). The smallest effect observed, between the PKDD and the MCS score (|r|= 0.30), is suggestive of discriminant validity. A linear model for known-groups validity demonstrated PGIS-stratified PKDD means increased with increased PGIS score, with a large effect size (η^2^ = 0.274), although mean PKDD scores were similar across PGIS categories 2–4 (from “Moderately” through to “Very much”) (Additional file 1: Table S9).

### Reliability and validity—PKDIA

Internal consistency was also high for the PKDIA, ω = 0.90, and test–retest reliability was excellent [30], *ICC* (2,1) = 0.87. Medium to large correlation were observed between the PKDIA and all co-validating scores (|r|= 0.50–0.82) (Fig. 4). As with the PKDD, the strongest correlation was observed with the FACT-An Additional Concerns Subscale (|r|= 0.82), showing evidence for convergent validity (Fig. 4). Known-groups validity demonstrated PGIS-stratified PKDIA means increased with increased PGIS score, with a large effect size (η^2^ = 0.508) (Additional file 1: Table S9).

## Discussion

This analysis assessed the psychometric performance of the first two PK deficiency-specific PRO instruments, the PKDD and PKDIA, using blinded data from the phase 3 ACTIVATE trial of mitapivat [15], the first approved targeted treatment of PK deficiency in adults [32].

Both the PKDD and PKDIA performed well across the psychometric analyses applied. Internal consistency was high for both the PKDD and PKDIA (ω = 0.86 and 0.90, respectively), with excellent test–retest reliability (*ICC* (2, 1) of 0.94 and 0.87). Convergent validity was established via small to medium (|r| of 0.30–0.73) and medium to large correlations (|r| of 0.50–0.82) between the PKDD and PKDIA and other co-validating scores. Although the FACT-An Additional Concerns Subscale had the strongest correlations with both the PKDD and PKDIA, these two instruments evaluate a broader range of signs, symptoms and impacts that better reflect the heterogeneous manifestation of PK deficiency beyond the symptoms and impacts of fatigue which are the focus of the FACT-An. The PKDD and PKDIA therefore provide patients with PK deficiency greater opportunities to detail valuable information on the specific aspects of the disease that cannot be replicated using FACT-An.

Baseline PGIS was selected for use as the known-groups set as it is a widely employed PRO that provides a simple 1-item outcome measure specifically focused on patient perception of the severity of their condition, with clearly defined categories of severity. A linear model for known-groups validity demonstrated PGIS-stratified PKDD and PKDIA means increased with increased PGIS score, as expected, with large effect sizes for both assessments. As both the PKDD and PKDIA also correlated well with PGIS, these results suggest that PGIS is a reasonable criterion measure for known-groups validity. These analyses provide evidence that the PKDD and PKDIA are valid and reliable PRO tools to measure disease impact, signs, and symptoms in adults with PK deficiency. Therefore, these are very likely to represent appropriate measures for the capture of relevant and informative feedback from patients on the severity of their disease and impact of treatment on their quality of life and symptoms. As such, the PKDD and PKDIA represent the first validated PK deficiency-specific PRO measures.

This analysis has several limitations. Validation was performed in-trial, which may restrict the spectrum of latent traits under investigation due to the selection criteria necessary in recruitment of a study population. Although the study population was substantial for this rare anemia, the small overall patient population does represent a limitation of this analysis despite high response rates to the surveys at the assessed timepoints. The known-groups validity analysis is somewhat limited by the use of a single known-groups set (PGIS) based on patient self-reports, and future research may examine additional sets that focus on other relevant patient characteristics to enhance the current psychometric analyses. It is also of note that the PKDD and PKDIA instruments were also designed for use in a clinical trial setting, which may limit their utility in a real-world clinical setting due to the complex scoring algorithms required, the expectation that the PKDD is completed at the end of the day (which may not align with patients’ clinician visits), the need for completion of the PKDD on at least four days of a seven-day period for stable scoring estimates, and the use of electronic deployment of the instruments throughout the trial which may not be available as part of standard care and may thus have enhanced the likelihood of reliable data capture in the trial. However, there may be utility in the use of the simple sum scores in a clinical setting, as while these do not provide a validated estimate, they may serve to give specialist clinicians an approximation of patient status [33].

## Conclusion

The results presented here provide evidence of validity and reliability for the first disease-specific PROs for the assessment of the symptoms and disease impact of PK deficiency in patients within a clinical trial setting, the PKDD and PKDIA, and support the PKDD and PKDIA as valid, reliable, and responsive tools to measure and evaluate changes in disease impact in clinical trials for adults with PK deficiency.

### Supplementary Information

**Additional file 1: Table S1.** Schedule of validation-related assessments. **Table S2.** Efficacy measures used for co-validation of the PKDD and PKDIA. **Table S3.** PKDD item response distributions at baseline. **Table S4.** PKDD and PKDIA model factor loading and item response model parameters. **Table S5.** PKDD θ means (SE) across baseline days. **Table S6.** PKDD scoring algorithm. **Table S7.** PKDIA item response distributions at baseline. **Table S8.** PKDIA scoring algorithm. **Table S9.** Linear model of baseline PKDD and PKDIA known-group validity, stratified by PGIS

## Data Availability

Qualified researchers may request access to related clinical study documents. Please send your data sharing requests to datasharing@agios.com. The following considerations will be taken into account as part of the review: 1. Ability for external researcher to re-identify trial participants such as small rare disease trials or single-center trials. 2. Language used in data and requested documents (eg, English or other). 3. Informed consent language with respect to allowance for data sharing. 4. Plan to re-evaluate safety or efficacy data summarized in the approved product labeling. 5. Potential conflict of interest or competitive risk.

## Abbreviations

CFI: Comparative fit index
CI: Confidence interval
df: Degrees of freedom
EAP: Expected a priori
EQ-5D-5L: European Quality of Life Five-Dimensional Descriptive System
EQ-5D-5L UK: United Kingdom-normed European quality of life 5-dimension score
EQ-5D-5L US: United States-normed European quality of life 5-dimension score
FACT-An: Functional Assessment of Cancer Therapy-Anemia
GRM: Graded response model
HRQoL: Health-related quality of life
ICC (2,1): 2-way mixed-effects intra-class correlation coefficient
IRT: Item-response theory
MCS: Mental component summary
NRS: Numerical rating scale
PCS: Physical component summary
PGIS: Patient global impression of severity
PK: Pyruvate kinase
PKDD: Pyruvate Kinase Deficiency Diary
PKDIA: Pyruvate Kinase Deficiency Impact Assessment
PKR: Pyruvate kinase R
PROs: Patient-reported outcomes
RBCs: Red blood cells
RMSEA: Root mean squared error of approximation
SD: Standard deviation
SF-12v2: 12-Item short form health survey version 2.0
SRMR: Standardized root mean square residual
TLI: Tucker–Lewis index
US FDA: United States Food and Drug Administration
VDS: Verbal descriptor scale
