# Supplementary material for: Psychometric validation of the Pyruvate Kinase Deficiency Diary and Pyruvate Kinase Deficiency Impact Assessment in adults in the phase 3 ACTIVATE trial
Source: J Patient Rep Outcomes. 2023 Nov 9;7:112. doi: 10.1186/s41687-023-00650-3 (PMC10636000; doi:10.1186/s41687-023-00650-3)
Supplement: Supplementary file 1 — Additional file 1: Table S1. Schedule of validation-related assessments. Table S2. Efficacy measures used for co-validation of the PKDD and PKDIA. Table S3. PKDD item response distributions at baseline. Table S4. PKDD and PKDIA model factor loading and item response model parameters. Table S5. PKDD θ means (SE) across baseline days. Table S6. PKDD scoring algorithm. Table S7. PKDIA item response distributions at baseline. Table S8. PKDIA scoring algorithm. Table S9. Linear model of baseline PKDD and PKDIA known-group validity, stratified by PGIS [file 41687_2023_650_MOESM1_ESM.docx]

# Supplementary materials and appendices

**Table S1** Schedule of validation-related assessments

**Table S2** Efficacy measures used for co-validation of the PKDD and PKDIA

**Table S3** PKDD item response distributions at baseline

**Table S4** PKDD and PKDIA model factor loading and item response model parameters

**Table S5** PKDD 𝜃 means (SE) across baseline days

**Table S6** PKDD scoring algorithm

**Table S7** PKDIA item response distributions at baseline

**Table S8** PKDIA scoring algorithm

**Table S9** Linear model of baseline PKDD and PKDIA known-group validity, stratified by PGIS

# Table S1 Schedule of validation-related assessments

| **Visit** | | | | | | | | | | | | | |
| --- | --- | --- | --- | --- | --- | --- | --- | --- | --- | --- | --- | --- | --- |
|  | **Screening period** | **Dose-optimization period (Part 1)** | | | | | | | | | **Fixed-dose period (Part 2)** | | |
|  | Screening | D1 | W2 | W3 | W4 | W6 | W7 | W8 | W10 | W12 | W16 | W20 | W24/End of study^a^ |
| Study day | D −42 to  D −1 | 1 | 15 | 22 | 29 | 43 | 50 | 57 | 71 | 85 | 113 | 141 | 169 |
| Visit window |  | 0 | ±3D | ±3D | ±3D | ±3D | ±3D | ±3D | ±3D | ±3D | ±3D | ±3D | ±4D |
| **HRQoL assessments^b^** | | | | | | | | | | | | | |
| EQ-5D-5L | X^c^ |  |  |  |  |  |  |  |  | X |  |  | X |
| SF-12v2 | X^c^ |  |  |  |  |  |  |  |  | X |  |  | X |
| PKDD | Daily | Daily throughout the 24-week study period | | | | | | | | | | | |
| PKDIA | X^c^ |  |  |  | X |  |  | X |  | X | X | X | X |
| FACT-An | X^c^ |  |  |  |  |  |  |  |  | X |  |  | X |
| PGIS | X^c^ |  |  |  | X |  |  | X |  | X |  |  | X |

^a^Subjects who discontinued the study at any time prior to the W24 visit were required to attend the end of study visit 28±4 days after the last study visit that the subject attended or 28±4 days after the last dose of study treatment, whichever was later.

^b^All subjects were given an eDiary (Signant Health ePRO platform) at screening to record responses to HRQoL assessments. The PKDD assessment was collected daily throughout Screening, Part 1, and Part 2. The eDiary assessments were expected to be completed by the subject in the evening within the window for the relevant study visit.

^c^PKDIA, FACT-An, SF12v2, EQ-5D-5L, and PGIS were expected to be completed on the first day of Screening (+1 day) and then at weekly intervals (±1 day) until Part 1 Day 1.

*D* day, *EQ-5D-5L* European quality of life 5-dimension score, *EQ-5D-5L US* United States-normed European quality of life 5-dimension score, *FACT-An* functional assessment of cancer therapy anemia, *HRQoL* health-related quality of life, *PGIS* patient global impression of severity, *PKDD* Pyruvate Kinase Deficiency Diary, *PKDIA* Pyruvate Kinase Deficiency Impact Assessment, *SF-12v2* 12-item short form health survey version 2, *W* week

# **Table S2** Efficacy measures used for co-validation of the PKDD and PKDIA

| **Measure** | | **Description** |  | **Score range** |
| --- | --- | --- | --- | --- |
| EQ-5D-5L | Evaluates patient QoL over 5 dimensions: mobility, self-care, usual activities, pain, and mood | | Collected on the first day of screening, at weekly intervals (up to 6 weeks) during screening, and then at Weeks 12 and 24 | 0–100 |
| FACT-An | A 20-item QoL measure which assesses fatigue and anemia-related concerns | | Collected on the first day of screening, at weekly intervals (up to 6 weeks) during screening, and then at Weeks 12 and 24 | 0–188 |
| PGIS | A disease-specific single item questionnaire which rates patient impression of the severity of their condition from “Not at all” to “Very much” | | Collected on the first day of screening, at weekly intervals (up to 6 weeks) during screening, and then every 4 weeks | 0–4  (Not at all–very much) |
| MCS-12 | Component of the SF-12v2 assessing mental health (phycological distress and well-being) on a continuous scale | | Collected on the first day of screening, at weekly intervals (up to 6 weeks) during screening, and then at Weeks 12 and 24 | 0–100 |
| PCS-12 | Component of the SF-12v2 assessing physical functioning on a continuous scale | | Collected on the first day of screening, at weekly intervals (up to 6 weeks) during screening, and then at Weeks 12 and 24 | 0–100 |

*EQ-5D-5L UK* United Kingdom-normed European quality of life 5-dimension score, *EQ-5D-5L US* United States-normed European quality of life 5-dimension score, *FACT-An* functional assessment of cancer therapy anemia, *MCS-12* 12-item mental component summary, *PCS-12* 12-item physical component summary, *PGIS* patient global impression of severity, *PKDD* Pyruvate Kinase Deficiency Diary, *PKDIA* Pyruvate Kinase Deficiency Impact Assessment, *QoL* quality of life, *SF12v2* 12-item short form healthy survey version 2.0

# Table S3 PKDD item response distributions at baseline

|  | **PKDD** | | **Item** | | | | | | | | | | | | | | | | | | | | | | |  | | | |  |
| --- | --- | --- | --- | --- | --- | --- | --- | --- | --- | --- | --- | --- | --- | --- | --- | --- | --- | --- | --- | --- | --- | --- | --- | --- | --- | --- | --- | --- | --- | --- |
|  | **Response** | | **0** | | **1** | | **2** | | **3** | | **4** | | **5** | | **6** | | **7** | | **8** | | **9** | | **10** | | **Missing data** | | | **Total** | | |
| Item 1 | Freq | | 4 | | 3 | | 2 | | 6 | | 4 | | 5 | | 13 | | 19 | | 4 | | 2 | | 1 | | 0 | | | 63 | | |
|  | % Valid | | 6.35 | | 4.76 | | 3.17 | | 9.52 | | 6.35 | | 7.94 | | 20.63 | | 30.16 | | 6.35 | | 3.17 | | 1.59 | | NA | | | 100 | | |
|  | % Total | | 6.35 | | 4.76 | | 3.17 | | 9.52 | | 6.35 | | 7.94 | | 20.63 | | 30.16 | | 6.35 | | 3.17 | | 1.59 | | 0 | | | 100 | | |
| Item 2 | Freq | | 6 | | 1 | | 2 | | 5 | | 5 | | 14 | | 14 | | 8 | | 4 | | 2 | | 2 | | 0 | | | 63 | | |
|  | % Valid | | 9.52 | | 1.59 | | 3.17 | | 7.94 | | 7.94 | | 22.22 | | 22.22 | | 12.70 | | 6.35 | | 3.17 | | 3.17 | | NA | | | 100 | | |
|  | % Total | | 9.52 | | 1.59 | | 3.17 | | 7.94 | | 7.94 | | 22.22 | | 22.22 | | 12.70 | | 6.35 | | 3.17 | | 3.17 | | 0 | | | 100 | | |
| Item 3 | Freq | | 11 | | 23 | | 22 | | 7 | | 0 | | — | | — | | — | | — | | — | | — | | 0 | | | 63 | | |
|  | % Valid | | 17.46 | | 36.51 | | 34.92 | | 11.11 | | 0 | |  | |  | |  | |  | |  | |  | | NA | | | 100 | | |
|  | % Total | | 17.46 | | 36.51 | | 34.92 | | 11.11 | | 0 | | — | | — | | — | | — | | — | | — | | 0 | | | 100 | | |
| Item 4^a^ | Freq | | 26 | | 6 | | 7 | | 2 | | 2 | | 3 | | 1 | | 0 | | 1 | | 0 | | 1 | | 14 | | | 63 | | |
|  | % Valid | | 53.06 | | 12.24 | | 14.29 | | 4.08 | | 4.08 | | 6.12 | | 2.04 | | 0 | | 2.04 | | 0 | | 2.04 | | NA | | | 100 | | |
|  | % Total | | 41.27 | | 9.52 | | 11.11 | | 3.17 | | 3.17 | | 4.76 | | 1.59 | | 0 | | 1.59 | | 0 | | 1.59 | | 22.22 | | | 100 | | |
| Item 5^b^ | Freq | | 14 | | 6 | | 5 | | 4 | | 4 | | 4 | | 6 | | 2 | | 3 | | 0 | | 0 | | 15 | | | 63 | | |
|  | % Valid | | 29.17 | | 12.50 | | 10.42 | | 8.33 | | 8.33 | | 8.33 | | 12.50 | | 4.17 | | 6.25 | | 0 | | 0 | | NA | | | 100 | | |
|  | % Total | | 22.22 | | 9.52 | | 7.94 | | 6.35 | | 6.35 | | 6.35 | | 9.52 | | 3.17 | | 4.76 | | 0 | | 0 | | 23.81 | | | 100 | | |
| Item  6 | | Freq | | 1 | | 5 | | 5 | | 8 | | 10 | | 10 | | 11 | | 6 | | 3 | | 4 | | 0 | | | 0 | | 63 | |
|  |  | % Valid | | 1.59 | | 7.94 | | 7.94 | | 12.70 | | 15.87 | | 15.87 | | 17.46 | | 9.52 | | 4.76 | | 6.35 | | 0 | | | NA | | 100 | |
|  |  | % Total | | 1.59 | | 7.94 | | 7.94 | | 12.70 | | 15.87 | | 15.87 | | 17.46 | | 9.52 | | 4.76 | | 6.35 | | 0 | | | 0 | | 100 | |
| Item  7 | | Freq | | 2 | | 2 | | 1 | | 8 | | 7 | | 9 | | 16 | | 5 | | 8 | | 3 | | 2 | | | 0 | | 63 | |
|  |  | % Valid | | 3.17 | | 3.17 | | 1.59 | | 12.70 | | 11.11 | | 14.29 | | 25.40 | | 7.94 | | 12.70 | | 4.76 | | 3.17 | | | NA | | 100 | |
|  |  | % Total | | 3.17 | | 3.17 | | 1.59 | | 12.70 | | 11.11 | | 14.29 | | 25.40 | | 7.94 | | 12.70 | | 4.76 | | 3.17 | | | 0 | | 100 | |

^a^Item 4 had a response option of “I have never experienced bone pain” that accounts for the 14 responses of missing data.

^b^Item 5 had response options of “I avoided this activity because it was too difficult for me to do moderate physical activity” and “NA, because I did not have the opportunity to do moderate physical activity” that account for the 15 responses of missing data.

The % Valid row represents the distribution proportions excluding missing data where optional response categories were not relevant or applicable to the patient. The % Total row presents the distribution proportions including any missing data.

See Table 1 for a full summary of PKDD items. In brief, item 1, tiredness at its worst; 2, tiredness after daily activities; 3, jaundice; 4, bone pain; 5, shortness of breath; 6, energy at the beginning of the day; 7, energy at the end of the day.

*Freq* frequency, *NA* not applicable, *PKDIA* Pyruvate Kinase Deficiency Impact Assessment

# Table S4 PKDD and PKDIA model factor loading and item response model parameters

| **PKDD^a^** | **λ** | **a1** | **d1** | **d2** | **d3** | **d4** | |
| --- | --- | --- | --- | --- | --- | --- | --- |
| Item 1 | 0.96 | 5.65 | 8.18 | 4.05 | 0.12 | −4.97 | |
| Item 2 | 0.96 | 5.65 | 8.18 | 4.05 | 0.12 | −4.97 | |
| Item 3 | 0.23 | 0.40 | 1.54 | −0.09 | −2.05 | −4.41 | |
| Item 4 | 0.40 | 0.75 | −0.74 | −1.85 | −2.74 | −4.17 | |
| Item 5 | 0.49 | 0.95 | 0.71 | −0.40 | −1.40 | −3.06 | |
| Item 6 | 0.54 | 1.08 | 2.68 | 1.02 | −0.62 | −2.42 | |
| Item 7 | 0.63 | 1.36 | 3.56 | 1.83 | 0.20 | −1.95 | |
| Fit statistics | C_2_(14) = 11.81, *p* = 0.622, RMSEA <0.01 (95% CI <0.001, 0.132), SRMR = 0.15,  TLI = 1.000, CFI = 1.00 | | | | | |  |
| **PKDIA^b^** | **λ** | **a1** | **d1** | **d2** | **d3** | **d4** | |
| Item 2 | 0.78 | 2.10 | 2.78 | 0.84 | −0.41 | −3.68 | |
| Item 5 | 0.88 | 3.10 | 1.94 | −0.24 | −1.93 | −5.85 | |
| Item 6 | 0.95 | 5.38 | 2.39 | 0.07 | −4.11 | −6.77 | |
| Item 7 | 0.97 | 6.83 | 3.55 | −0.17 | −4.37 | −8.36 | |
| Item 8 | 0.86 | 2.86 | 0.54 | −1.08 | −2.66 | −5.52 | |
| Item 10 | 0.75 | 1.90 | 1.40 | −0.12 | −1.64 | −4.02 | |
| Item 11 | 0.79 | 2.17 | 1.80 | −0.05 | −1.91 | −3.49 | |
| Item 12 | 0.77 | 2.04 | 2.92 | 1.29 | −1.00 | −3.26 | |
| Fit statistics | C_2_(20) = 22.28, *p* = 0.326, RMSEA = 0.043 (95% CI <0.001, 0.120), SRMR = 0.09,  TLI = 1.000, CFI = 1.00 | | | | | |  |

Model fit success criteria were defined as: C_2_ *p* value >0.05; RMSEA ≈ 0.05 and <0.1; SRMR <0.1; TLI and CFI >0.9.

^a^Four assumptions were selected through model fitting: 1. due to concerns regarding the parsing of a low number of items into smaller subscales items were fitted as single factors to ensure maintenance of factor replicability (Gorsuch 1983); 2. the collapsed response sets were used to allow identification of the model; 3. factor structure (IRT parameters of slope and intercepts) were assumed to be consistent across days so that the model structure held for responses across the baseline week; 4. items 1 and 2 had their item parameters constrained to be equal to better stabilize the model fit, due to similarity in the first model’s fit.

^b^Four items were not included for scoring following model fits and evaluation of item performance within modelling procedures. Item 1: high redundancy with Item 2 with less preferable item response parameters; Items 3 and 4: demonstrated very large floor effects and high inter-item correlation (r = 0.77), and were indicated as misfitting within the model and therefore potentially lacked relevance for this patient population; Item 9: low rate of relevance to patients for Item 9a.

See Table 1 for a full summary of PKDD and PKDIA items. In brief: PKDD item 1, tiredness at its worst; 2, tiredness after daily activities; 3, jaundice; 4, bone pain; 5, shortness of breath; 6, energy at the beginning of the day; 7, energy at the end of the day. PKDIA item 1, starting things you want to get done; 2, finishing things you want to get done; 3, bothered by appearance; 4, unwanted attention; 5, impact on household activities; 6, impact on social activities; 7, impact on leisure activities; 8, impact on social relationships; 9, impact on work/school; 10, concentration; 11, physical activity; 12, additional sleep or rest.

*λ* factor loading, *a1* item slope, *C_2_* test statistic for absolute model fit, *CFI* comparative fit index, *CI* confidence interval, *d1–d4* item intercepts, *PKDD* Pyruvate Kinase Deficiency Diary, *PKDIA* Pyruvate Kinase Deficiency Impact Assessment, *RMSEA* root mean squared error of approximation, *SRMR* standardized root mean square residual, *TLI* Tucker-Lewis index

# Table S5 PKDD 𝜃 means (SE) across baseline days

| **Day** | **Mean 𝜃 (SE)** | **Variance 𝜃 (SE)** |
| --- | --- | --- |
| −1 | 0 (N/A) | 1 (N/A) |
| −2 | 0.11 (0.143) | 1.13 (0.273) |
| −3 | 0.08 (0.151) | 1.06 (0.262) |
| −4 | 0.01 (0.157) | 1.31 (0.325) |
| −5 | −0.02 (0.146) | 1.22 (0.263) |
| −6 | 0.01 (0.150) | 1.21 (0.280) |
| −7 | −0.06 (0.116) | 0.73 (0.234) |

*𝜃* latent variable, *PKDD* Pyruvate Kinase Deficiency Diary, *SE* standard error

# Table S6 PKDD scoring algorithm

| **Step** | **Information** |
| --- | --- |
| 1 | Scoring includes items 1–7 |
| 2 | For items 6 and 7 (𝑚_6𝑗_ and 𝑚_7𝑗_, for study day 𝑗, respectively) reverse key the values according to:  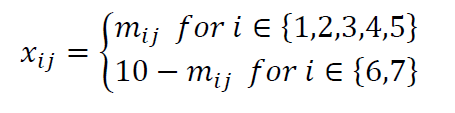 |
| 3 | Collapse response options for items 1, 2, 4, 5, 6, and 7 (𝑥_𝑖_) according to:  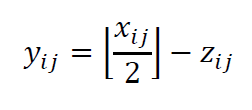  Where  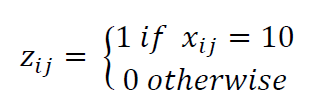  The data should look like this mapping:  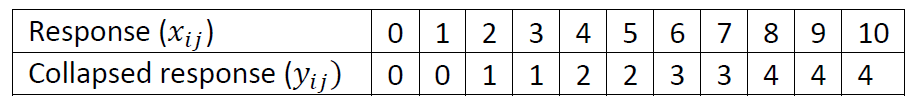  For each item (𝑖) on each administration of the PKDD (𝑗) |
| 4 | Sum the observed collapsed items (𝑦_𝑖_) for each administration (𝑗) across study weeks:  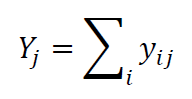  For all observed item ratings (𝑖). |
| 5 | Merge the scoring table (see below) onto the data by the variable sum score (𝑌_𝑗_); the scoring table is organized by sum score. T-scores are to be used as the PKDD daily scores: |
| 6 | Determine the weekly score (𝑇_𝑤_) according to the following calculation by averaging daily scores (𝑇_𝑗_) for at least four administration days (𝑑) within a given study week (𝑤):  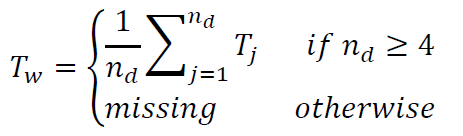  Where total administrations (𝑛_𝑑_ ∈ [0,7]) is the number of administrations (𝑗) for a given patient within a given study week (𝑤) based on study day (𝑑):  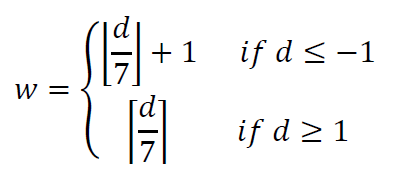 |

| Sum Score | T-Score |
| --- | --- |
| 0 | 25 |
| 1 | 27 |
| 2 | 29 |
| 3 | 32 |
| 4 | 34 |
| 5 | 36 |
| 6 | 38 |
| 7 | 40 |
| 8 | 42 |
| 9 | 43 |
| 10 | 45 |
| 11 | 47 |
| 12 | 48 |
| 13 | 50 |
| 14 | 52 |
| 15 | 53 |
| 16 | 55 |
| 17 | 57 |
| 18 | 59 |
| 19 | 61 |
| 20 | 63 |
| 21 | 64 |
| 22 | 66 |
| 23 | 68 |
| 24 | 69 |
| 25 | 71 |
| 26 | 73 |
| 27 | 75 |
| 28 | 76 |

*PKDD* Pyruvate Kinase Deficiency Diary

# Table S7 PKDIA item response distributions at baseline

|  | **PKDIA** | **Item** | | | | | | | | | |  | |  | |  | |  | |  |
| --- | --- | --- | --- | --- | --- | --- | --- | --- | --- | --- | --- | --- | --- | --- | --- | --- | --- | --- | --- | --- |
|  | **Response** | **0** | **1** | **2** | **3** | **4** | **5** | **6** | **7** | **8** | **9** | **10** | **Missing data** | | **QA-1** | | **QA-2** | | **Total** | |
| Item 1 | Freq | 8 | 4 | 7 | 5 | 12 | 6 | 11 | 16 | 7 | 1 | 1 | 0 | |  | |  | | 78 | |
|  | % Valid | 10.26 | 5.13 | 8.97 | 6.41 | 15.38 | 7.69 | 14.10 | 20.51 | 8.97 | 1.28 | 1.28 | 0 | |  | |  | | 100 | |
|  | % Total | 10.26 | 5.13 | 8.97 | 6.41 | 15.38 | 7.69 | 14.10 | 20.51 | 8.97 | 1.28 | 1.28 | 0 | |  | |  | | 100 | |
| Item 2 | Freq | 9 | 3 | 10 | 6 | 7 | 8 | 8 | 21 | 3 | 2 | 1 | 0 | |  | |  | | 78 | |
|  | % Valid | 11.54 | 3.85 | 12.82 | 7.69 | 8.97 | 10.26 | 10.26 | 26.92 | 3.85 | 2.56 | 1.28 | 0 | |  | |  | | 100 | |
|  | % Total | 11.54 | 3.85 | 12.82 | 7.69 | 8.97 | 10.26 | 10.26 | 26.92 | 3.85 | 2.56 | 1.28 | 0 | |  | |  | | 100 | |
| Item 3 | Freq | 20 | 12 | 8 | 6 | 3 | 4 | 8 | 5 | 6 | 1 | 5 | 0 | |  | |  | | 78 | |
|  | % Valid | 25.64 | 15.38 | 10.26 | 7.69 | 3.85 | 5.13 | 10.26 | 6.41 | 7.69 | 1.28 | 6.41 | 0 | |  | |  | | 100 | |
|  | % Total | 25.64 | 15.38 | 10.26 | 7.69 | 3.85 | 5.13 | 10.26 | 6.41 | 7.69 | 1.28 | 6.41 | 0 | |  | |  | | 100 | |
| Item 4 | Freq | 27 | 14 | 5 | 5 | 5 | 8 | 6 | 1 | 4 | 2 | 1 | 0 | |  | |  | | 78 | |
|  | % Valid | 34.62 | 17.95 | 6.41 | 6.41 | 6.41 | 10.26 | 7.69 | 1.28 | 5.13 | 2.56 | 1.28 | 0 | |  | |  | | 100 | |
|  | % Total | 34.62 | 17.95 | 6.41 | 6.41 | 6.41 | 10.26 | 7.69 | 1.28 | 5.13 | 2.56 | 1.28 | 0 | |  | |  | | 100 | |
| Item 5 | Freq | 16 | 6 | 9 | 10 | 7 | 9 | 11 | 7 | 1 | 2 | 0 | 0 | |  | |  | | 78 | |
|  | % Valid | 20.51 | 7.69 | 11.54 | 12.82 | 8.97 | 11.54 | 14.10 | 8.97 | 1.28 | 2.56 | 0 | 0 | |  | |  | | 100 | |
|  | % Total | 20.51 | 7.69 | 11.54 | 12.82 | 8.97 | 11.54 | 14.10 | 8.97 | 1.28 | 2.56 | 0 | 0 | |  | |  | | 100 | |
| Item 6 | Freq | 16 | 8 | 7 | 6 | 15 | 10 | 4 | 5 | 4 | 3 | 0 | 0 | |  | |  | | 78 | |
|  | % Valid | 20.51 | 10.26 | 8.97 | 7.69 | 19.23 | 12.82 | 5.13 | 6.41 | 5.13 | 3.85 | 0 | 0 | |  | |  | | 100 | |
|  | % Total | 20.51 | 10.26 | 8.97 | 7.69 | 19.23 | 12.82 | 5.13 | 6.41 | 5.13 | 3.85 | 0 | 0 | |  | |  | | 100 | |
| Item 7 | Freq | 19 | 3 | 6 | 10 | 5 | 16 | 6 | 6 | 5 | 2 | 0 | 0 | |  | |  | | 78 | |
|  | % Valid | 24.36 | 3.85 | 7.69 | 12.82 | 6.41 | 20.51 | 7.69 | 7.69 | 6.41 | 2.56 | 0 | 0 | |  | |  | | 100 | |
|  | % Total | 24.36 | 3.85 | 7.69 | 12.82 | 6.41 | 20.51 | 7.69 | 7.69 | 6.41 | 2.56 | 0 | 0 | |  | |  | | 100 | |
| Item 8 | Freq | 25 | 8 | 8 | 8 | 6 | 8 | 7 | 5 | 2 | 0 | 1 | 0 | |  | |  | | 78 | |
|  | % Valid | 32.05 | 10.26 | 10.26 | 10.26 | 7.69 | 10.26 | 8.97 | 6.41 | 2.56 | 0 | 1.28 | 0 | |  | |  | | 100 | |
|  | % Total | 32.05 | 10.26 | 10.26 | 10.26 | 7.69 | 10.26 | 8.97 | 6.41 | 2.56 | 0 | 1.28 | 0 | |  | |  | | 100 | |
| Item 9^a^ | Freq | 9 | 1 | 5 | 7 | 4 | 11 | 7 | 7 | 3 | 0 | 0 |  | | 3 | | 21 | | 78 | |
|  | % Valid | 16.67 | 1.85 | 9.26 | 12.96 | 7.41 | 20.37 | 12.96 | 12.96 | 5.56 | 0 | 0 |  | |  | |  | | 100 | |
|  | % Total | 11.54 | 1.28 | 6.41 | 8.97 | 5.13 | 14.10 | 8.97 | 8.97 | 3.85 | 0 | 0 |  | | 3.85 | | 26.92 | | 100 | |
| Item 10 | Freq | 20 | 2 | 7 | 10 | 6 | 13 | 4 | 12 | 1 | 2 | 1 | 0 | |  | |  | | 78 | |
|  | % Valid | 25.64 | 2.56 | 8.97 | 12.82 | 7.69 | 16.67 | 5.13 | 15.38 | 1.28 | 2.56 | 1.28 | 0 | |  | |  | | 100 | |
|  | % Total | 25.64 | 2.56 | 8.97 | 12.82 | 7.69 | 16.67 | 5.13 | 15.38 | 1.28 | 2.56 | 1.28 | 0 | |  | |  | | 100 | |
| Item 11^b^ | Freq | 9 | 7 | 10 | 6 | 10 | 7 | 5 | 4 | 3 | 1 | 1 |  | | 6 | | 9 | | 78 | |
|  | % Valid | 14.29 | 11.11 | 15.87 | 9.52 | 15.87 | 11.11 | 7.94 | 6.35 | 4.76 | 1.59 | 1.59 |  | |  | |  | | 100 | |
|  | % Total | 11.54 | 8.97 | 12.82 | 7.69 | 12.82 | 8.97 | 6.41 | 5.13 | 3.85 | 1.28 | 1.28 |  | | 7.69 | | 11.54 | | 100 | |
| Item 12 | Freq | 11 | 14 | 26 | 19 | 8 |  |  |  |  |  |  |  | | 0 | |  | | 78 | |
|  | % Valid | 14.10 | 17.95 | 33.33 | 24.36 | 10.26 |  |  |  |  |  |  |  | | 0 | |  | | 100 | |
|  | % Total | 14.10 | 17.95 | 33.33 | 24.36 | 10.26 |  |  |  |  |  |  |  | | 0 | |  | | 100 | |

^a^For Item 9a, QA-1 = No, because it was too difficult for me to go to work or school, and QA-2 = No, because I am not currently working or in school for reasons unrelated to my PK deficiency. 9b was recorded only for patients who answered “yes” to 9a.

^b^For Item 11a, QA-1 = No, because it was too difficult for me to do moderate physical activity, and QA-2 = No, because I did not have the opportunity to do moderate physical activity. 11b was recorded only for patients who answered “yes” to 11a.

The % Valid row presents the distribution proportions excluding missing data where optional response categories were not relevant or applicable to the patient from the denominator. The % Total row presents the distribution proportions including any missing data.

See Table 1 for a full summary of PKDIA items. In brief, item 1, starting things you want to get done; 2, finishing things you want to get done; 3, bothered by appearance; 4, unwanted attention; 5, impact on household activities; 6, impact on social activities; 7, impact on leisure activities; 8, impact on social relationships; 9, impact on work/school; 10, concentration; 11, physical activity; 12, additional sleep or rest.

*Freq* frequency, *NA* not applicable, *PKDIA* Pyruvate Kinase Deficiency Impact Assessment, *QA* question

# Table S8 PKDIA scoring algorithm

| **Step** | **Information** |
| --- | --- |
| 1 | Scoring includes items 2, 5–8, 10, 11b, and 12 |
| 2 | Collapse response options for items 2, 5, 6, 7, 8, 10, and 11b (𝑥_𝑖_) according to:  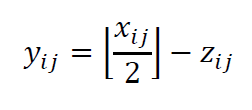  Where  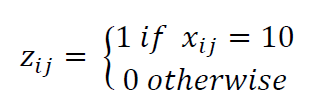  The data should look like this mapping:  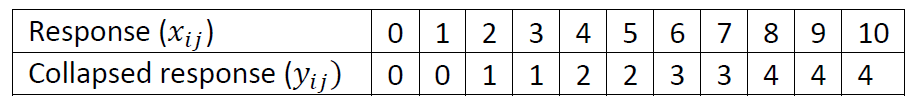  For each item (𝑖) on each administration of the PKDIA (𝑗) |
| 3 | Sum the collapsed items (𝑦_𝑖_) plus item 12 (𝑥_12_) for each administration (𝑗):  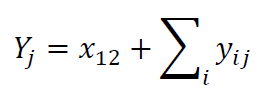 |
| 4 | Merge the scoring table (see below) onto the data by the variable sum score (𝑌_𝑗_); the scoring table is organized by sum score. T-scores are to be used as the PKDIA scores: |

| Sum Score | T-Score |
| --- | --- |
| 0 | 30 |
| 1 | 35 |
| 2 | 37 |
| 3 | 39 |
| 4 | 40 |
| 5 | 42 |
| 6 | 43 |
| 7 | 44 |
| 8 | 45 |
| 9 | 46 |
| 10 | 47 |
| 11 | 48 |
| 12 | 49 |
| 13 | 50 |
| 14 | 51 |
| 15 | 52 |
| 16 | 53 |
| 17 | 54 |
| 18 | 55 |
| 19 | 56 |
| 20 | 57 |
| 21 | 58 |
| 22 | 59 |
| 23 | 60 |
| 24 | 61 |
| 25 | 62 |
| 26 | 63 |
| 27 | 64 |
| 28 | 66 |
| 29 | 67 |
| 30 | 69 |
| 31 | 72 |
| 32 | 76 |

*PKDIA* Pyruvate Kinase Deficiency Impact Assessment

# Table S9 Linear model of baseline PKDD and PKDIA known-group validity, stratified by PGIS

|  | **PKDD** | | | |
| --- | --- | --- | --- | --- |
| **PGIS** | **n** | **Mean** | **SD** | 𝜼^𝟐^ |
| 0 | 14 | 41.3 | 10.5 | 0.274 |
| 1 | 27 | 47.6 | 5.2 |  |
| 2 | 16 | 53.8 | 6.2 |  |
| 3 | 14 | 53.0 | 3.8 |  |
| 4 | 1 | 52.4 | — |  |
| NA^a^ | 1 | 42.5 | — |  |
|  | **PKDIA** | | | |
| **PGIS** | **n** | **Mean** | **SD** | 𝜼^𝟐^ |
| 0 | 13 | 39.8 | 7.6 | 0.508 |
| 1 | 27 | 45.8 | 6.5 |  |
| 2 | 17 | 54.7 | 5.6 |  |
| 3 | 12 | 57.3 | 5.1 |  |
| 4 | 2 | 60.5 | 7.8 |  |
| NA^a^ | 7 | 45.3 | 9.4 |  |

^a^Patients with missing data at the specified analysis visit.

*𝜼^𝟐^* partial regression coefficient, *NA* not applicable, *PGIS* patient global impression of severity, *PKDD* Pyruvate Kinase Deficiency Diary, *PKDIA* Pyruvate Kinase Deficiency Impact Assessment, *SD* standard deviation
